# Formal Thought Disorder and Self-Disorder: An Empirical Study

**DOI:** 10.3389/fpsyt.2021.640921

**Published:** 2021-04-08

**Authors:** Julie Nordgaard, Mette Gravesen-Jensen, Marlene Buch-Pedersen, Josef Parnas

**Affiliations:** ^1^Mental Health Centre Amager, Copenhagen, Denmark; ^2^Department of Clinical Medicine, University of Copenhagen, Copenhagen, Denmark; ^3^Early Psychosis Intervention Centre, Psychiatry East, Roskilde, Denmark; ^4^Mental Health Centre Glostrup, Copenhagen, Denmark; ^5^Center for Subjectivity Research, University of Copenhagen, Copenhagen, Denmark

**Keywords:** schizophrenia, schizotypy, proverb, bizarre, literal, self-disorder, formal thought disorder

## Abstract

**Background:** Formal thought disorder was constitutively linked to the original concept of schizophrenia and has since been one of central features supporting its diagnosis. Bleuler considered formal thought disorder as a fundamental symptom of schizophrenia among other fundamental symptoms, including ego disorders. The contemporary concept of self-disorder represents a more developed, nuanced, and systematic approach to disturbances of self-experience than the Bleulerian concept of ego disorders. As fundamental symptoms, on Bleuler's account, are persistently present in every case, an association between these symptoms could be expected. The purpose of this study was to examine the association between self-disorder and formal thought disorder.

**Methods:** A sample of 94 diagnostically heterogeneous patients was examined for formal thought disorder using clinical rating and a proverb test. The proverb test was analyzed for two different aspects of formal thought disorder: literal responses and bizarre responses. The sample was comprehensively assessed for psychopathology, including self-disorder as measured with the Examination of Anomalous Self-Experience scale.

**Results:** The patients, who provided bizarre responses, had a higher level of self-disorder, more negative symptoms, lower level of social functioning, and lower level of intelligence. Bizarre answers aggregated in patients diagnosed within the schizophrenia spectrum compared with patients outside the schizophrenia spectrum. We found moderate correlations between the two measures of formal thought disorder (clinically rated and bizarre responses) and self-disorder (0.454 [*p* < 0.01] and 0.328 [*p* < 0.01]). Literal responses did not differ between diagnostic groups and also did not correlate with bizarre responses. Specificity of bizarre responses for a diagnosis within schizophrenia spectrum was 86.89%, whereas sensitivity was 40.85%.

**Conclusion:** The close relation between formal thought disorder and self-disorder further adds to the notion of self-disorder as a unifying psychopathological core beneath the apparently heterogeneous symptoms of schizophrenia.

## Introduction

Formal thought disorder was constitutive for the creation of the concept of schizophrenia. Kraepelin (1) used the term “Zerfahrenheit,” which is sometimes translated into “incoherence,” but which also refers to more subtle distortions of meaning in the patient's speech (2). Bleuler also used this term, but he introduced the general notion of “loosening of associations” as a fundamental symptom of schizophrenia (3). On his account, the fundamental symptoms were specific for the diagnosis of schizophrenia. Formal thought disorder has been subsequently included in most definitions of schizophrenia, although with permutations of its characteristic features and severity. Importantly, the notion of formal thought disorder must be distinguished from the broad Anglo-Saxon concept of “thought disorder.” The latter term is more inclusive and refers to a variety of disturbance of thought, including disorders of thought content such as delusions.

In current literature, it is frequent to use the term “speech disorder.” Obviously, we only have access to the patients' thought processes through their expressions, in speech, writing, or behavior [e.g., “unsinnige Handlungen” (4, 5)]. *Formal* thought disorder refers to disturbances in the structure (or *form*) of thinking, e.g., the boundaries of concept formation, semantic disturbances (such as neologisms), or disturbances in the transitions between thought segments (6, 7). Although formal thought disorder is expressed through the patient's speech, it is often difficult to detect disorder of thinking, if the patient's responses are brief or laconic or in interviews with a high degree of structure. It is a well-established clinical observation that the more structured the conversation is, the less prominent the manifestation of the patient's formal thought disorder will be (6). Thus, there are basically two approaches to detect and measure formal thought disorder. One, used in all clinical assessments, is simply the rating of disorder manifest in the patient's speech. Nancy Andreasen has published a detailed scale of “thought, language, and communication disorder” (8) that can be used for this approach. Another approach is based in the tradition of psychological projective tests such as object sorting test or Rorschach (e.g., Holzman's Thought Disorder Index) (9). Another possible test, which is one we also used in the current study, is the proverb test in which the patient is presented with a number of different proverbs, which he then must explain the meaning of (see below) (10).

Studies of formal thought disorder have shown that formal thought disorder may be considered a marker of illness severity. Basic demographics do not seem to be related to the presence of formal thought disorder, whereas the level of intelligence has been related to bizarre thinking. Studies examining the association between social functioning and formal thought disorder have shown ambiguous results (7, 11).

As already mentioned, Bleuler considered formal thought disorder as a fundamental symptom of schizophrenia related to other fundamental symptoms such as ego disorders. However, in modern literature, the relationship between disturbances of self-experience and formal thought disorder has not been probably explored except from theoretical suggestions (12, 13). [For details of self-disorder, see, e.g., (14, 15)]. In the current study, we were interested in the following questions:

1. How is self-disorder as measured by the Examination of Anomalous Self-Experience (EASE) (16) related to clinically rated formal thought disorder and formal thought disorder rated through proverb test?
2. How is formal thought disorder related to other canonical dimensions of schizophrenia (positive and negative symptoms) as well as to social functioning?

## Materials and Methods

### Sample

The original sample comprised the first 100 admissions, diagnostically heterogeneous patients aged between 18 and 65 years. The sample comprised the consecutive first admissions to the Psychiatric Center Hvidovre (a psychiatric inpatient facility of the University of Copenhagen) that provides psychiatric service to a population of 150,000 in one particular catchment area of the City of Copenhagen (there are no private inpatient psychiatric facilities in Denmark). The patients were included over a period of 18 months independently of their clinical diagnosis at admission. All consecutive first admissions were screened for eligibility. If there were more eligible patients than it was possible to examine within the pragmatic constraints of the project, the youngest patient was always selected. The patients participated on the condition of informed consent, and a relevant medical ethical committee approved the study.

The patients had to be in a condition in which they could tolerate a lengthy interview, because one of the goals of the primary study was to assess the adequacy of different psychopathological interviews (17). This led to exclusion of aggressive, agitated, or severely psychotic patients, who were not able to collaborate. Additional exclusion criteria comprised primary or clinically dominating substance use, history of brain injury, and organic brain disorder. Involuntarily admitted or legal patients were also excluded. Moreover, all participants had to have an intelligence level within the normal range as measured by the Intelligenz-Struktur Test 2000 R (18). All patients were asked to do a proverb test, but four patients declined. After inclusion in the project, two patients later withdrew their consent to the research project. Thus, a total of 94 patients took the proverb test.

### Assessments

Formal thought disorder was assessed using a proverb test consisting of 11 proverbs. The test was chosen as it is easy to administer, not too time consuming, and it is a commonly used test for rating bizarre responses (19, 20). We analyzed the proverbs for (1) literalness and (2) bizarreness, as these are two commonly agreed aspects of formal thought disorder (21). For literalness, we followed the scoring manual by Hertler et al. (22). Based on selected keywords in the proverbs, it was assessed whether these keywords were attributed literalness. We only looked for presence or absence of literalness. To illustrate the test: the proverb “a thief believes everybody steals” means that one ascribes to others one's own flawed or weak mindset or habit. Here are two examples of responses that were rated for literalness: “if you yourself are a thief, you assume that all people are thieves too,” and “people who break the law believe that it is normal.”

For rating bizarreness, we constructed a simple scoring system for bizarre answers inspired by Exner's comprehensive system for scoring the Rorschach (23), more specifically, the DV (Deviant Response) score. Again, we only looked for presence or absence of bizarre response. We rated answers as bizarre; if they were idiosyncratic; if the rater was unable, or found it difficult, to grasp the meaning of the answer; or if there was a private use of terms or expressions in which the meaning may be clear, but the expression itself is unusual. The rating was done jointly and based on clinical judgment in the same way as one would approach the scoring of DV in a Rorschach protocol. Here are a few examples of responses, which we rated bizarre in the present sample: Asked to explain the meaning of the proverb “don't cry over spilt milk,” which means that there is no reason to get upset over something that have already occurred and that cannot be changed, one patient responded, “One should not get upset. To have courage or to get courage.” Another example, asked to explain the meaning the proverb “many a mickle makes a muckle,” which means that lots of small amounts can be accumulated to large amount, one patient responded, “that means that you have to stay young.”

### Raters of Formal Thought Disorder

All ratings of formal thought disorder (i.e., literal and bizarre responses to the proverbs) were done by two psychologists (MBP and MGJ), both of which have vast clinical experience and extensive training and experience with the use of the Rorschach test. Both raters were blinded to diagnosis, any kind of psychopathological information, and level of functioning of the patients.

### Psychopathology and Diagnosis

All patients were thoroughly assessed for psychopathology and diagnosed by JN and JP. The interviews were split over two to three sessions, and the total duration of the interviews was 3–6 h. The interview was carried out by an experienced psychiatrist and expert in the use and teaching of the EASE (16) (JN). The interviews were conducted in a semistructured, conversational style, including a thorough psychosocial history, a description of the illness evolution, the Operational Criteria Checklist (OPCRIT) (24) expanded with additional items from the Schedule for Affective Disorders and Schizophrenia (SADS) (25), the Examination of Anomalous Self-Experiences (EASE) (16), the perceptual section from the Bonn Scale for the Assessment of Basic Symptoms (BSABS) (26), and of abnormal expressive features (27). The proverb test was administered after completed interviews. All interviews were videotaped.

The present study used lifetime *International Classification of Diseases, 10th Revision* (*ICD-10*) diagnoses based on all available, diagnostically relevant information (interview videos, notes, information from the hospital charts, which also contained second informant descriptions of the illness' symptoms and their evolution). Self-disorder was assessed using the EASE and rated on a lifetime basis as present or absent. We constructed a “positive symptom scale” (including psychotic symptoms) and a “negative symptom scale” (including negative symptoms) by adding items selected from the interview schedule in order to obtain the measures of the canonical dimensions of schizophrenic symptomatology. Table 1 shows the composition and Cronbach α's of the positive and negative symptoms scales in addition to a scale of items targeting clinically rated formal thought disorder.

**Table 1:** Psychopathological scales.

**Positive symptom scale**	**Negative symptom scale**
Hallucinations	Disturbance of volition, avolition, inertia
Delusions	Apathy
Persecutory delusions	Social withdrawal
Delusional grandiosity	Anergy
	Alogia, poverty of speech
Chronbach α = 0.710	Cronbach α = 0.721
**Clinically rated formal thought disorder**	
Incoherence	
Tendency to idiosyncratic or bizarre communication	
Rapport compromised by formal thought disorder	
Tangentiality	
Illogical thinking	
Cronbach α = 0.709	

The variable “social and professional difficulties” was created by summing two items from the interview checklist: “social difficulties” (which covered difficulties in personal relationships as measured by <2 close relations) and “professional difficulties” (which covered difficulties in maintaining jobs or education within the last year); maximal score was 2.

### Ethics

All patients participated upon written consent. The study was approved by the Danish Data Protection Agency. According to Danish legislation, approval from The Danish National Committee on Health Research Ethics is not required for interview studies of this kind. The study adhered to the ethical principles laid down by the Helsinki Declaration.

### Statistical Analysis

We analyzed the sample divided into two groups: one group, consisting of patients who provided at least one bizarre response, and another group of patients, who did not provide any bizarre response.

Subsequently, we did some extra analyses with the sample divided into two diagnostic groups: schizophrenia spectrum disorders (i.e., schizophrenia, other non-affective psychoses, and schizotypy) and non-schizophrenia spectrum disorders (including bipolar disorder, major depression, personality disorder, anxiety, and adjustment disorder).

We tested for equality of means by *t* test when normally distributed and Mann–Whitney *U* test when not. Correlations were tested with Spearman ρ. For these analyses, we used SPSS version 26. Specificity and sensitivity were calculated using Medcalc's diagnostic test calculator (28).

## Results

### Literal Responses

Table 2 shows that the number of patients with literal responses did not differ between the two groups (bizarre responses vs. no bizarre responses). When dividing the sample into two groups depending on diagnosis, i.e., schizophrenia spectrum disorders vs. all other disorders, we found no significant differences between the groups for literal responses. Looking at the whole sample, we did not find any significant correlation between self-disorder and literal responses to the proverbs.

**Table 2:** Sample description, psychopathology, and diagnostic groups.

	**Bizarre response**	**Non-bizarre response**	**Statistics**
*n*	32	62	
Gender (male/female)	11/21	20/42	*T* test *p* = 0.838
Mean age	27.1 (sd = 7.7)	28.0 (sd = 9.9)	*T* test *p* = 0.640
Social and professional difficulties, mean	1.50 (sd = 2.35)	0.50 (sd = 0.64)	Mann–Whitney *p* = 0.003
Literal answers to proverbs	0.5 (sd = 0.508)	0.32 (sd = 0.471)	*T* test *p* = 0.096
Positive symptoms, mean	4.25 (sd = 0.68)	3.10 (sd = 3.99)	Mann–Whitney *p* = 0.078
Negative symptoms, mean	2.00 (sd = 1.50)	1.29 (sd = 1.50)	Mann–Whitney *p* = 0.022
Clinically assessed formal thought disorder, mean	2.22 (sd = 2.2)	0.74 (sd = 1.33)	Mann–Whitney *p* = 0.000
Total EASE score, mean	19.56 (sd = 7.93)	13.81 (sd = 8.90)	Mann–Whitney *p* = 0.002
Intelligence level (max is 80), mean	32.35 (sd = 10.91)	39.24 (sd = 12.52)	*T* test *p* = 0.011
Diagnostic group			
Schizophrenia	18	25	
Schizotypal disorder	11	17	
Other mental illness	3	20	
	**Schizophrenia spectrum disorders**	**All other disorders**	**Statistics**
*n*	71	23	
At least one literal answer (*n*)	27 (38%)	9 (39%)	χ^2^ = 0.009 (*p* = 0.925)
At least one bizarre answer (*n*)	29 (41%)	3 (13%)	χ^2^ = 5.788 (*p* = 0.016)

*The sample is divided in two different ways*.

*sd, standard deviation*.

### Bizarre Responses

Table 2 shows significant differences between the group with bizarre responses and the group with no bizarre responses in regard to self-disorder, negative symptoms, clinically rated formal thought disorder, social and professional difficulties, and level of intelligence. We tested if intelligence was a mediating factor for the effect of self-disorder on bizarre responses by linear regression and found that it was not. Self-disorder and level of intelligence were independently contributing to bizarre responses.

The correlations between bizarre responses and other variables are displayed in Table 3. The correlation between self-disorder (mean EASE score) and bizarre responses was moderate (ρ = 0.328, *p* < 0.01). Bizarre responses significantly aggregated in the schizophrenia spectrum disorders group.

**Table 3:** Correlation matrix Spearman ρ.

	**Bizarre responses**	**Literal responses**	**Positive symptoms**	**Clinically rated formal thought disorders**	**Negative symptoms**	**EASE total score**	**Level of intelligence**
Bizarre responses	—						
Literal responses	0.178	—					
Positive symptoms	0.181	0.107	—				
Clinically rated formal thought disorder	0.398^**^	0.136	0.310^**^	—			
Negative symptoms	0.222^*^	−0.002	0.378^**^	0.230^*^	—		
EASE total score	0.328^**^	0.153	0.317^**^	0.454^**^	0.489^**^	—	
Level on intelligence	−0.265^*^	−0.177	−0.294^**^	−0.142	0.005	−0.040	—

** *Correlation is significant at the 0.01 level (two-tailed)*.

* *Correlation is significant at the 0.05 level (two-tailed)*.

### Schizophrenia Spectrum Disorders vs. All Other Diagnoses

At the bottom of Table 2, it can be seen that 29 of 71 patients within the schizophrenia spectrum gave at least one bizarre response to the proverbs. By contrast, only three patients with a diagnosis outside the schizophrenia spectrum gave a bizarre response.

Finally, we found that the specificity of bizarre responses for schizophrenia spectrum diagnoses was 86.89%, whereas the sensitivity was 40.85% (Table 4).

**Table 4:** Diagnostic sensitivity and specificity of bizarre responses for a schizophrenia spectrum disorder.

	**Value**	**95% confidence interval**
Sensitivity	40.85%	29.32–53.16%
Specificity	86.89%	66.41–97.22%
Positive likelihood ratio	3.13	1.05–9.33
Negative likelihood ratio	0.68	0.53–0.87
Positive predictive value	90.38%	75.92–96.55%
Negative predictive value	32.89%	27.62–38.61%
Accuracy	52.37%	41.81–62.78%

## Discussion

This is the first study to systematically examine the relation between formal thought disorder and self-disorder. We found a highly significant, moderate correlation between bizarre responses and self-disorder. The correlation between bizarre answers and negative symptoms as well as clinically rated formal thought disorder was significant, whereas the correlation between bizarre answers and positive symptoms was not. Moreover, we found significantly more difficulties in social and occupational functioning in the group of patients who had given a bizarre response. These findings point to bizarre responses tapping into something central for schizophrenia spectrum disorders (i.e., schizophrenia, other non-affective psychoses, and schizotypy).

Literal responses did not relate significantly with any of the examined variables and did not differ between the diagnostic groups. Literal and bizarre responses did not correlate with each other and appear to reflect different dimensions. These findings are in line with previous studies, which have shown that the formal thought disorder of idiosyncratic verbalizations, autistic logic, and absurd thinking are more characteristic of schizophrenia (9, 29–31).

The specificity of bizarre answers for a diagnosis within schizophrenia spectrum disorders was high, suggesting that the presence of formal thought disorder should prompt clinicians to suspect or at least examine if the patient may fulfill the criteria for a schizophrenia spectrum disorder. Conversely, the absence of formal thought disorder cannot rule out schizophrenia spectrum disorders, as a considerable proportion of these patients did not offer bizarre responses. However, it should be emphasized that these figures should be taken with caution because the confidence intervals in Table 4 vary considerably, and schizophrenia spectrum diagnoses are partly dependent on the presence of formal thought disorder.

From a theoretical point of view, the association between expressive features (formal thought disorder) and subjective experiences (e.g., self-disorder) is perhaps not so surprising. In general, formal thought disorders are typically conceived as a disturbance in the individual's capacity of producing and expressing thoughts in terms of semantics or relations between semantic units. Alternatively, formal thought disorder can be seen as the individual's ability to draw upon the intersubjective resources that help structure our thinking and expression (note that these two views are not mutually exclusive). In the latter view, we have to distinguish, in the terms of Merleau-Ponty, between “le langue” and “la parole” (32). The former is a historically and socially determining intersubjective matrix that dictates or influences our conceptual/cognitive abilities. “Le langue” is this matrix, whereas “la parole” is the individual's thinking and speech. In Merleau-Ponty's view, cognition is therefore heavily dependent on perception and embodied interaction in the social world, in agreement with earlier views of Vygotski (33). In the case of schizophrenia, it has repeatedly been emphasized that consciousness here entails a sort of disintegration. Kraepelin considered the disintegration as a product of weakened center of self-consciousness (Ich-Bewusstsein) (34). Famously, Bleuler introduced the concept of “loosening of associations” as an instance of a general tendency of splitting (Spaltung). Unfortunately, Bleuler's view was often understood as a sort of mechanical deficit in associative mechanisms. Bleuler himself, however, saw this splitting as a lack of hierarchy in the goal-directedness or intentionality. As an example of this intentionality, he mentions a peasant, whose overarching goal is to maximize the productivity of his land. Other activities such as sowing or plowing are subordinate activities to his primary goal. The peasant may do other things such as eating or sleeping, but the overarching goal is always tacitly present in his mind and structures his behavior. Thus, Bleuler saw the splitting and loosening of associations as an expression of diminished intentional directedness of consciousness. He ascribed this deficiency to a disorder of the ego and its activity (3). In the psychoanalytic literature of ego psychology, disorder of thinking was also considered as an expression of the pathology of the self (35).

Viewed from the perspective of the EASE-based self-disorder research, we can point to the following aspects of the link between self-disorder and formal thought disorder. First, self-disorder implies a weakened intersubjective attachment (a disorder of “common sense”), a tendency to solipsistic experiences and generally unstable self-awareness leading to a confusion between modalities of intentionality (14, 16, 36). Self-disorder may also imply a range of preverbal experiences, which may be quite unique and unusual and create a difficulty for conceptualization and verbalization, perhaps prompting the patient to use private or idiosyncratic formulations to articulate prereflectively altered self-experience. Finally, patients with schizophrenia spectrum disorder often seem to exist in different and sometimes competing ontological frameworks, one reflecting our natural attitude and one more private where the laws of causality and non-contradiction do not exist (37–39).

In this study, we approached the issue of self-disorder from a different perspective than that of diagnoses, as the purpose was to explore the association between formal thought disorder and self-disorder. Our findings seem to converge around central phenomena of schizophrenia spectrum disorders, including self-disorder. From our own and others' studies, we know that self-disorder constitutes a trait phenomenon that is present before the full symptomatology of schizophrenia manifests (40–44), suggesting that self-disorder constitutes a basic framework within which the heterogeneous symptoms associated with schizophrenia may be unified and to some extent understood.

In the diagnostic systems of *ICD-10* and *Diagnostic and Statistical Manual of Mental Disorders, Fifth Edition*, formal thought disorder is defined at a quite severe level; perhaps leading to more subtle manifestations of formal thought disorder tends to be overlooked. Our findings point to the importance of paying close attention to potential formal thought disorder in the clinical encounter. Additionally, clinicians must be aware that interviews with a high degree of structure can impede formal thought disorder from materializing, and obviously assessments using self-rating scales do not allow for tracking formal thought disorder.

The major limitation to the study is the relatively small sample size. However, studies with such comprehensive assessment of psychopathology are very time consuming, making it difficult to obtain larger samples. Moreover, it should be mentioned that the translation of formally disturbed responses from Danish to English has built-in difficulties, and it is likely that some of the disturbed answers have lost some relevant aspects in the translation.

## Data Availability Statement

The datasets generated for this article are not readily available because it contains sensitive personal information. Requests to access the datasets should be directed to Julie_nordgaard@dadlnet.dk.

## Ethics Statement

Ethical review and approval was not required for the study on human participants in accordance with the local legislation and institutional requirements. The patients/participants provided their written informed consent to participate in this study.

## Author Contributions

JN and JP designed the study and JN collected the data. MG-J and MB-P did the analyses. JN wrote the first draft of the manuscript. All authors contributed to the final manuscript.

## Conflict of Interest

The authors declare that the research was conducted in the absence of any commercial or financial relationships that could be construed as a potential conflict of interest.
